# Traumatic brain injury and autophagy: a pilot study about the immunohistochemical expression of LC3B, Beclin 1, p62, and LAMP2A in human autoptic samples

**DOI:** 10.3389/fnmol.2025.1562954

**Published:** 2025-04-28

**Authors:** Tommaso Livieri, Letizia Alfieri, Emiliana Giacomello, Djordje Alempijević, Tijana Petrovic, Yanko Georgiev Kolev, Davide Radaelli, Margherita Neri, Stefano D’Errico

**Affiliations:** ^1^Department of Medical Surgical and Health Sciences, University of Trieste, Trieste, Italy; ^2^Department of Medical Sciences, Section of Legal Medicine, University of Ferrara, Ferrara, Italy; ^3^Institute of Forensic Medicine “Milovan Milovanovic”, Faculty of Medicine, University of Belgrade, Belgrade, Serbia; ^4^Department of General Medicine, Forensic Medicine and Deontology, Medical University - Pleven, Pleven, Bulgaria

**Keywords:** traumatic brain injury, autophagy, immunohistochemistry, forensic, LC3B, Beclin 1, p62, LAMP2A

## Abstract

**Introduction:**

Autophagy is a cellular stress response that has been shown in the literature to be active in cerebral cells after a traumatic brain injury (TBI). The aim of this study is to investigate the potential use of four proteins involved in autophagy (LC3B, Beclin 1, p62, and LAMP2A), as a forensic diagnostic marker for TBI.

**Methods:**

We analyzed histological samples obtained from the frontal lobe of 10 subjects who died within 1 h of a TBI (Group A), 13 who died between 1 h and 32 days post-TBI (Group B), and a control group of 10 subjects who died without head trauma (Group C). Immunohistochemical (IHC) staining using anti-LC3B, anti-Beclin 1, anti-p62 and anti-LAMP2A antibodies was performed.

**Results and discussion:**

The results show that LC3B staining was the only one that show a statistically significant difference between groups. In particular, the percentage of neurons displaying an autophagic pattern was calculated from six random acquisitions per subject, and the results were compared across groups using one way ANOVA. Significant differences were observed between Groups A and B, and between Groups B and C, with *p*-values of 0.0055 and 0.0035, respectively. While the difference between Groups A and C was not statistically significant (*p*-value of 0.9845). These findings suggest that LC3B may serve as a useful diagnostic marker for TBI in cases where death is not immediate and open the door for further research.

## 1 Introduction

Autophagy is a cellular response to stress, that allows the controlled degradation of individual cellular organelles or, in extreme cases, the entire cell. This process encompasses three distinct pathways: macroautophagy, microautophagy, and chaperone-mediated autophagy (CMA) (Kobayashi, 2015). In macroautophagy, double-membrane vesicles, known as autophagosomes, encapsulate cellular components through a series of steps before fusing with lysosomes to facilitate their degradation (Yamamoto and Matsui, 2024). In contrast, microautophagy and CMA involve distinct molecular mechanisms that target cellular components for direct phagocytosis by lysosomes (Yao and Shen, 2023; Wang et al., 2023). The controlled degradation mediated by autophagy plays a fundamental role in maintaining organismal homeostasis. It recycles cellular waste into resources for metabolism and removes damaged or dysfunctional organelles, sometimes leading to programmed cell death under conditions of extreme starvation. This gradual degradation of cellular components ensures a safe pathway for surrounding tissues, serving as an alternative to apoptosis. Autophagy has a crucial role in maintaining homeostasis and responding to stress in numerous diseases, including Alzheimer’s disease, Parkinson’s disease, myopathies, cystic fibrosis, cardiac hypertrophy, infections, and obesity (Morishita and Mizushima, 2019; Mizushima and Levine, 2020; Lei and Klionsky, 2021; Yamamoto et al., 2023).

Traumatic Brain Injury (TBI) refers to brain damage caused by a traumatic event and is one of the leading causes of death and disability worldwide (Haarbauer-Krupa et al., 2021). In young people, motor vehicle accidents or sports injuries are the primary causes, while accidental falls are the main cause in older adults. TBI can be classified into primary injury, caused by an external force directly acting on the head, and secondary injury, resulting from the progression of the lesion through mechanisms such as ischemia and edema (Capizzi et al., 2020; Brazinova et al., 2021). However, post-mortem diagnosis and interpretation of TBI can be particularly challenging. Since the symptomatology is largely clinical, there is often little or no macroscopic or histological evidence of damage in the event of the victim’s death, especially without a complete description of the circumstances of the death or in cases of mild trauma (Bertozzi et al., 2020; Zwirner et al., 2022; Chen et al., 2022).

Nevertheless, literature indicates that autophagy is active in cerebral cells following TBI, functioning as a defense mechanism against neuronal damage (Clark et al., 2008; Wu and Lipinski, 2019; Zeng et al., 2020; Lang et al., 2024). Consequently, proteins involved in the three autophagy pathways could serve as diagnostic markers for TBI in forensic pathology, as well as prognostic tools in clinical practice.

A previous review by this group investigated on protein markers of autophagy in TBI models (Livieri et al., 2022). The review included all articles published up to May 27, 2022, that contained terms related to both autophagy and TBI. It identified 21 studies analyzing the alteration of 24 autophagy markers. The models used were predominantly mice and rats, with only one study conducted on human tissue (surgical samples from patients undergoing decompressive craniotomy). However, the study on human samples examined only two markers (LC3B and Beclin 1), and produced limited results (Clark et al., 2008). The techniques employed in these studies varied, predominantly involving immunofluorescence (IF) and western blot (WB), with only five studies utilizing immunohistochemistry (IHC) on 10 out of the identified 24 markers.

Based on multiple studies that reported variations in their expression following TBI in murine models, among the 24 markers identified in our review (Livieri et al., 2022), Lipidated Microtubule-Associated Protein Light-Chain 3B (LC3B), Beclin 1, p62 or Seqeuestrsomoe-1 (SQSTM1) and Lysosomal-Associated Membrane Protein 2A (LAMP2A) were selected for further analyses due to their promising potential. Aiming at finding a TBI marker for the forensic pathologist, the present study investigated the differences in IHC staining pattern of selected autophagy markers in neurons, between subjects who died from TBI and those who died from other causes. Understand whether the interval between the TBI and the death could have an impact on the modulation of these proteins pattern staining pattern was also considered.

## 2 Materials and methods

### 2.1 Case and sample selection

A retrospective case–control study was conducted on samples from subjects selected in the database of the Legal Medicine Departments of Cattinara Hospital in Trieste and the Unit of Legal Medicine of University of Ferrara. All subjects died between 2007 and 2024. The case group included 23 subjects who died within 32 days after suffering a TBI event. These subjects were divided into two groups: Group A consisted of those whose death was estimated to have occurred within 1 h after the trauma, and Group B included subjects whose death occurred between 1 h and 32 days after the trauma. As controls (Group C), 10 subjects who died of non-agonic causes, without significant head trauma or relevant pathologies, and with negative toxicological examinations were selected from the same databases.

All samples were collected during autopsies, ordered by the criminal prosecutor, carried out up to a maximum of 3 days after death. All the corpses included in the study were stored in a suitable refrigerated environment (4°C), after 24 h from death, until the necrosection was carried out. Once the tissue samples were collected, they were appropriately preserved in 10% buffered formalin solution and fixed for a period of no less than 2 weeks. The fixed samples were then embedded in paraffin. After sampling, each case and each control were allocated to one of the three groups and anonymized, assigning an alpha-numeric code.

For each case and for each control, histological samples of brain tissue were taken from the frontal lobe; if the frontal lobe cannot be sampled due to extensive destruction of the parenchyma, sampling was carried out from parietal lobe. Data of cases and controls are summarized in Table 1.

**Table 1:** Data of cases and controls.

N.	Sex	Age (y)	Interval between trauma and death (d)	Death cause
A1	M	52	0	Fall from height
A2	M	61	0	Head gunshot
A3	M	47	0	Head penetrant body
A4	M	38	0	Head gunshot
A5	M	83	0	Plane crash
A6	M	57	0	Head gunshot
A7	M	41	0	Head gunshot
A8	M	64	0	Head gunshot
A9	M	71	0	Road trauma
A10	F	52	0	Road trauma
B1	M	48	0^1^	Blunt force trauma
B2	M	58	1	Fall from height
B3	M	23	1	Blunt force trauma
B4	M	56	1	Blunt force trauma and fall from standing
B5	M	88	2	Fall from standing
B6	M	18	2	Sport trauma
B7	F	45	1	Road trauma
B8	F	67	22	Road trauma
B9	M	44	7	Fall from height
B10	M	83	18	Road trauma
B11	F	61	4	Road trauma
B12	M	50	14	Road trauma
B13	M	39	4	Blunt force trauma
C1	M	40	N/A	Hemorrhagic shock
C2	M	31	N/A	Chest gunshot
C3	M	40	N/A	Chest stab wound
C4	F	50	N/A	Arrhythmic sudden death
C5	M	40	N/A	Aortic dissection
C6	F	57	N/A	Atlanto-occipital dislocation
C7	M	40	N/A	Chest trauma
C8	M	27	N/A	Chest trauma
C9	M	27	N/A	Chest gunshot
C10	M	13	N/A	Atlanto-occipital dislocation

1 The case B1’s death occurred between 1 h and 1 days after the trauma.

### 2.2 Immunohistochemistry staining

Sections with a thickness of 4 μm were obtained from formalin-fixed paraffin-embedded samples using a microtome. After the deparaffinization and the rehydration, conducted as detailed in Table 2, the sections were pretreated for antigen retrieval with Diva Decloaker (Biocare Medical^®^, Pacheco, CA, United States) for 30 min in boiling water (100°C) and, after that, the endogenous peroxidases were blocked with 200 μL of Peroxide Block (Biocare Medical^®^, Pacheco, CA, United States) for 5 min at room temperature.

**Table 2:** Program of deparaffinization and hydration.

Reagent	Time
Xylene	30 min
Xylene	30 min
Alcohol 100%	30 min
Alcohol 100%	30 min
Alcohol 90%	5 min
Alcohol 70%	5 min
Distilled water	5 min
Distilled water	5 min

Then the sections were stained using MACH 1 Universal HRP-Polymer Detection kit (Biocare Medical^®^, Pacheco, CA, United States) according to the manufacturer’s protocol as shown in Table 3. Briefly, sections were blocked with Background Sniper to prevent unspecific binding, incubated with primary antibody and washed extensively. The concentration of primary antibody was determined after a titration experiment as 1:300 for anti-LC3B antibody (# GTX127375, GeneTex^®^, Irvine, CA, United States), 1:200 for anti-Beclin 1 antibody (# GTX31722, GeneTex^®^, Irvine, CA, USA), 1:500 for anti-p62 antibody (# GTX100685, GeneTex^®^, Irvine, CA, United States) and 1:300 for anti-LAMP2A antibody (# ab125068, abcam, Cambridge, United Kingdom). Specimens were then incubated with HRP-conjugated polymer, which recognizes antibodies raised in rabbit, and the reaction was developed with Diaminobenzidine. Finally, the slides were counterstained with hematoxylin for 2 min and dehydrated in ethanol 100%, diaphanized in xylene, and then mounted in resinous medium.

**Table 3:** MACH 1 universal HRP-polymer detection kit (Biocare Medical^®^, Pacheco, CA, United States) protocol.

Reagent	Quantity	Time	Temperature
Background sniper	200 μL	15 min	Room temperature
Primary antibody	200 μL	120 min	Room temperature
Mach1 universal HRP-Polymer	200 μL	30 min	Room temperature
3,3′-Diaminobenzidine	200 μL	5 min	Room temperature

The specificity of the primary antibodies was tested on positive controls, using sample of elderly patient with sarcopenia, as well as technical negative controls were included in the analyses. For the latter, two cases and two controls were casually selected and one slide of each was treated only with HRP-conjugated polymer without primary antibody (Supplementary Figure S1).

### 2.3 Specimen analysis

For a quantitative evaluation, for each specimen, an operator randomly acquired six fields at 40x magnification in the gray matter of the cerebral cortex, in a blinded manner. The operator then counted the number of neurons in each field and determined the percentage of neurons exhibiting an autophagic pattern using ImageJ software,^1^ using the “Manual Cell Counting and Marking” protocol of this software for RGB color, single, not stack images (accessed on 10 September 2024).^2^ Only intact and clearly recognizable neuronal somas with distinct nuclear and cytoplasmic components were considered.

Quantifications were expressed as the number of positive-stained cells/analyzed area. Histological analyses were performed by researchers who were blind to the information about the cases. The data were kept blinded until the analysis was terminated.

### 2.4 Statistical analysis

For the proteins that demonstrate a morphologic difference between groups (in LC3B and p62), data collected from the count were compiled in a database using GraphPad Prism version 10.00, (GraphPad Software, La Jolla, CA, United States) for statistical analysis. The average percentage of positive neurons was calculated for each group. To determine statistical differences among groups, data have been subjected to a normality test, and analyzed with a one-way ANOVA test followed by a post-hoc Tukey’s test.

## 3 Results

Specimens were analyzed as described in paragraph 2.3.

### 3.1 Evaluation of LC3B staining

**Figure 1:**
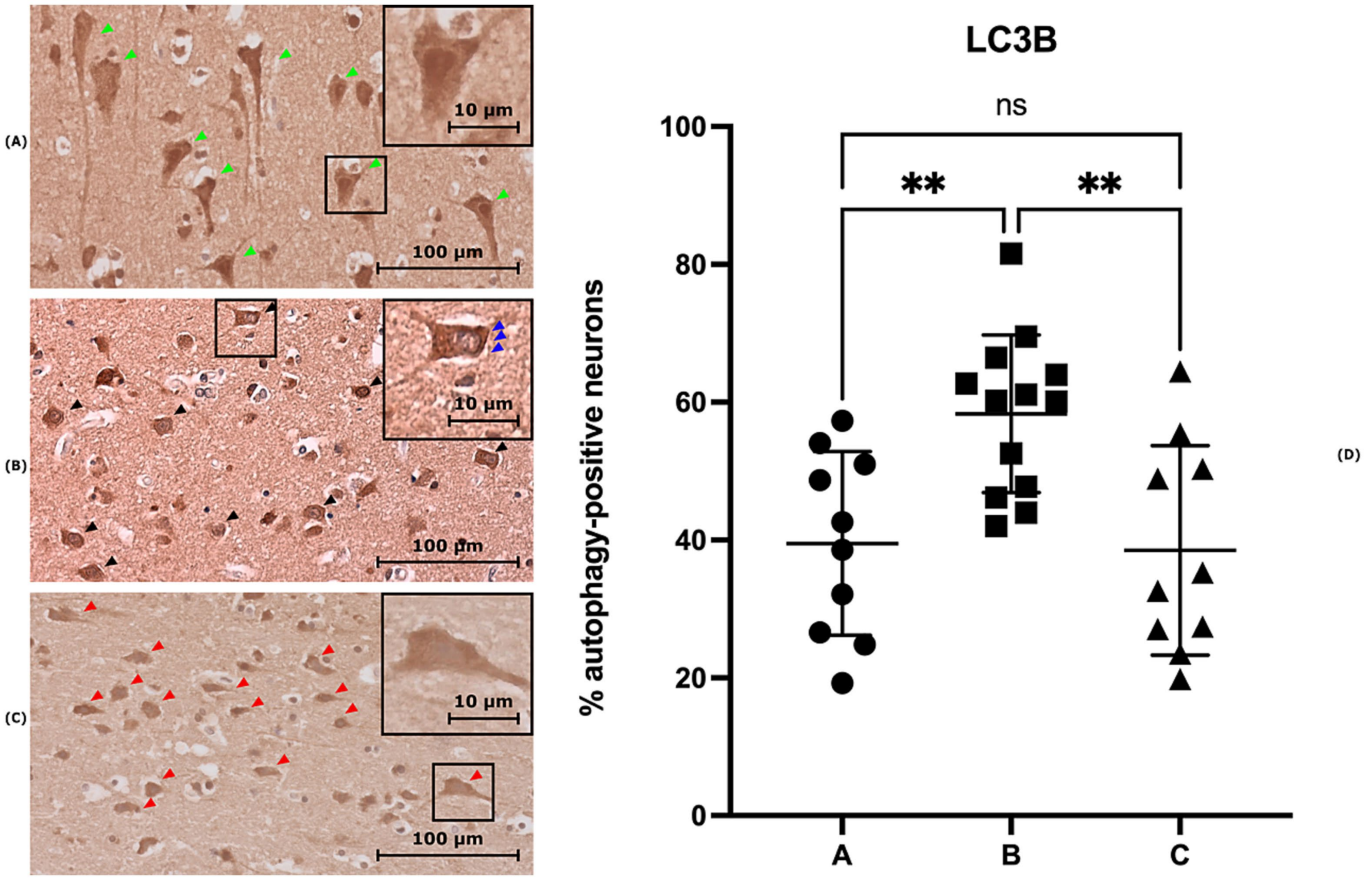
Three brain sections stained with anti-LC3B antibody. **(A)** Section from a case of Group A showing neurons with diffuse cytoplasmic staining (green arrows). **(B)** Section from a case of Group B, displaying neurons with staining indicative of an ongoing autophagic process (black arrows), characterized by vesicular cytoplasmic staining and perinuclear reinforcement (blue arrows). **(C)** Section from a control sample (group C), showing neurons with diffuse cytoplasmic staining (red arrows). **(D)** Distribution of mean percentage of neurons exhibiting an autophagic pattern in LC3B staining for each group (**p < 0.01).

The preliminary observation of the slides evidences a difference in the staining pattern between neurons that allow to differentiate an autophagic from a non-autophagic pattern. In particular, the autophagic pattern was defined as a vesicular cytoplasmic staining associated with perinuclear reinforcement, typically where the endoplasmic reticulum is located, indicating an ongoing autophagic process. This pattern is illustrated in Figure 1B, while non-autophagic neurons with diffuse cytoplasmic staining are shown in Figures 1A,C. Results issuing from neuron counts for each subject are presented as the mean percentage of neurons exhibiting an autophagic pattern in each field for each subject (Table 4).

**Table 4:** Mean percentage of neurons exhibiting an autophagic pattern in each field for each subject using LC3B staining.

Group A		Group B		Group C	
Subject N.	Positive neurons (%)	Subject N.	Positive neurons (%)	Subject N.	Positive neurons (%)
A1	19.26	B1	46.15	C1	50.32
A2	38.61	B2	62.72	C2	23.53
A3	32.16	B3	69.55	C3	48.94
A4	42.60	B4	43.94	C4	55.46
A5	54.04	B5	81.56	C5	19.81
A6	48.67	B6	64.00	C6	64.49
A7	24.81	B7	61.11	C7	27.46
A8	57.29	B8	42.00	C8	35.31
A9	50.98	B9	52.59	C9	32.63
A10	26.57	B10	47.74	C10	27.09
		B11	60.10		
		B12	66.48		
		B13	60.18		

As reported in Figure 1D, the one-way ANOVA between groups A and B (subjects whose death was estimated to have occurred within 1 h after the trauma and subjects whose death occurred between 1 h and 32 days after the trauma, respectively) as well as between groups B and C (controls), shows a significant difference (*p*-values of 0.0055 and 0.0035, respectively). In contrast, the difference between groups A and C was not statistically significant (*p*-value of 0.9845).

### 3.2 Evaluation of Beclin 1 staining

Beclin 1 staining resulted in a vesicular pattern diffused throughout all the cytoplasm. An extensive examination of the stained slides revealed no significant differences in the staining pattern in Beclin 1 expression between the study groups, as illustrated in Supplementary Figure S2. Due to the absence of discernible variations, this protein was not subjected to a statistical evaluation.

### 3.3 Evaluation of p62 staining

**Figure 2:**
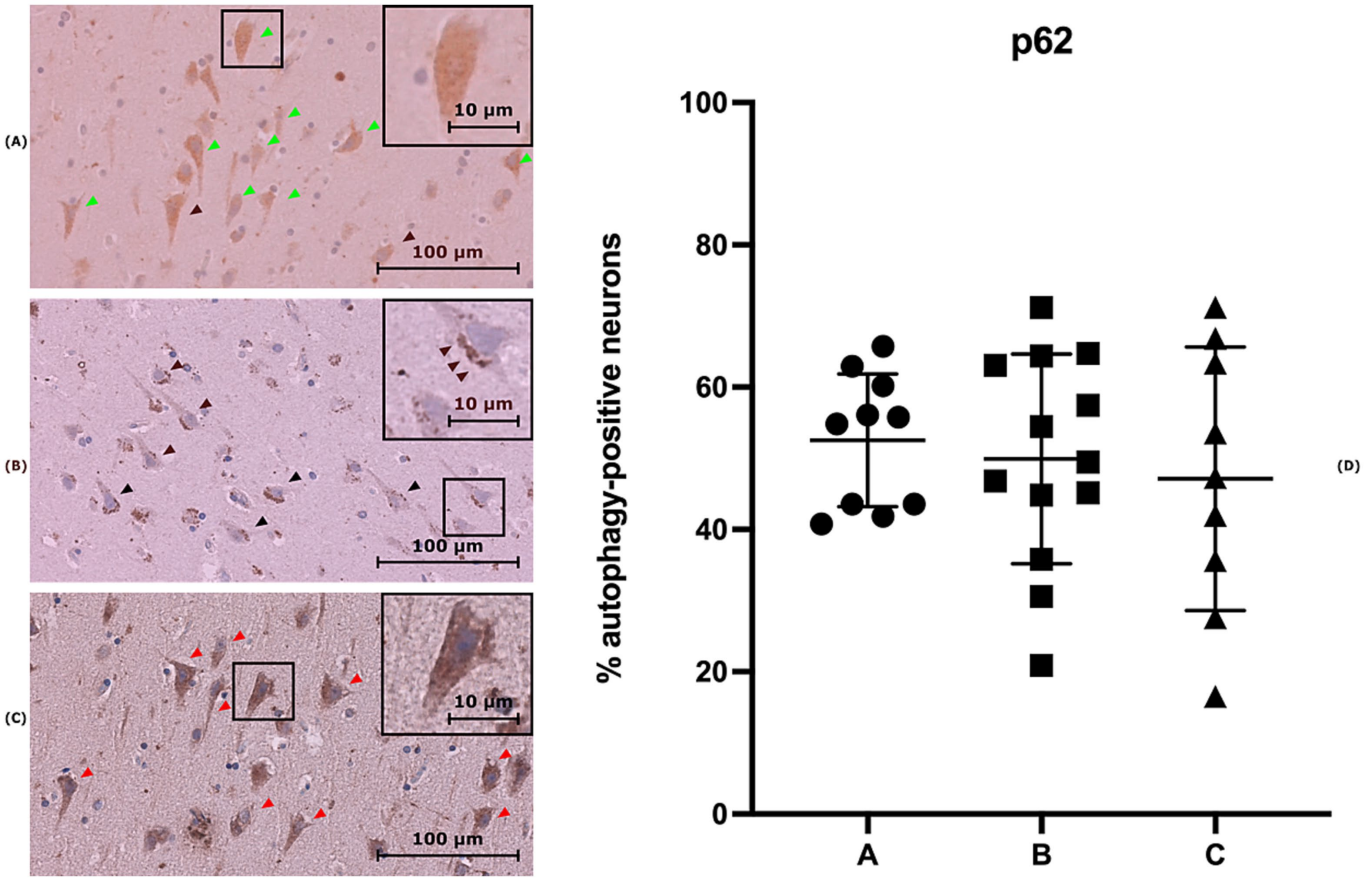
Three brain sections stained with anti-p62 antibody. **(A)** Section from a case of Group A showing neurons with diffuse cytoplasmic staining (green arrows). **(B)** Section from a case of Group B, displaying neurons with staining potentially indicative of an ongoing autophagic process (black arrows), characterized by vesicular pattern localized to a single pole of the cytoplasm (blue arrows). **(C)** Section from a control sample (group C), showing neurons with diffuse cytoplasmic staining (red arrows). **(D)** Distribution of mean percentage of neurons exhibiting an autophagic pattern in p62 staining for each group (**p < 0.01).

As LC3B staining, the preliminary examination of the slides revealed a difference in the staining pattern in neurons. Specifically, two distinct staining patterns were identified: a vesicular pattern localized to a single pole of the cytoplasm, as illustrated in Figure 2B, and a non-autophagic homogeneous pattern, as shown in Figures 2A,C. The neuron count results for each subject are expressed as the mean percentage of neurons exhibiting an autophagic pattern per field (Table 5).

**Table 5:** Mean percentage of neurons exhibiting an autophagic pattern in each field for each subject using p62 staining.

Group A		Group B		Group C	
Subject N.	Positive neurons (%)	Subject N.	Positive neurons (%)	Subject N.	Positive neurons (%)
A1	62.94	B1	30.59	C1	27.61
A2	56.07	B2	49.44	C2	41.97
A3	43.55	B3	35.82	C3	35.64
A4	40.77	B4	46.81	C4	63.40
A5	65.74	B5	20.90	C5	47.32
A.6	54.82	B6	54.47	C6	66.84
A7	41.84	B7	64.72	C7	53.56
A8	55.76	B8	64.38	C8	71.20
A9	60.20	B9	63.06	C9	N/A^1^
A10	43.56	B10	71.18	C10	53.18
		B11	57.45		
		B12	45.15		
		B13	44.80		

1 The case C9 was not stained with anti-p62 antibody because of the depletion of the sample.

The statistical analysis of p62 staining revealed no significant differences among the three groups, as shown in Figure 2D.

### 3.4 Evaluation of LAMP2A staining

The analysis of the slides showed a predominantly negative staining in neurons, while glial cells exhibited positive staining, as illustrated in Supplementary Figure S3. Since the focus of this study is on neuronal evaluation, no qualitative assessment was performed for LAMP2A.

## 4 Discussion

TBI refers to brain damage caused by a traumatic event, which is one of the leading causes of death and disability worldwide (Haarbauer-Krupa et al., 2021). However, post-mortem diagnosis and interpretation of TBI can be particularly challenging, because, at present, there is little or no macroscopic feature or histological parameter that can be useful in the diagnosis (Bertozzi et al., 2020; Zwirner et al., 2022; Chen et al., 2022). This evidence calls for the search of specific indicators, among which the literature suggests autophagic markers. Based on previous literature, the present work aimed at laying the background to identify possible autophagic markers in the post-mortem diagnosis of TBI in forensic samples.

LC3B is an autophagy protein involved in macroautophagy, particularly in the conjugation phase of autophagic vesicle formation (Yoshii and Mizushima, 2017). It is one of the most studied markers of autophagy also in TBI. Studies on murine models indicate that LC3B level increase within the first few hours after trauma and can remain elevated for up to 32 days, with a peak around 24 h post-trauma. Specifically, these studies show positivity in neurons during the first 3 days after trauma, followed by a shift to glial cells, with a peak on the third day post-trauma (Erlich et al., 2006; Lai et al., 2008; Liu et al., 2008; Zhang et al., 2008; Sadasivan et al., 2008; Sun et al., 2014; Sebastiani et al., 2017; Zhang et al., 2017).

Beclin-1 is part of the activation complex of macroautophagy and is the human equivalent of the yeast protein Atg6. Its action is inhibited by bcl-2. It was the first marker of autophagy that had been searched in post-TBI brains to prove the correlation between TBI and autophagy (Erlich et al., 2006; Diskin et al., 2005). Studies on murine models indicate an increase in Beclin 1 levels between 4 h and 32 days post-trauma, with a peak at approximately 24 h (Erlich et al., 2006; Zhang et al., 2008; Diskin et al., 2005).

The increase of the two proteins was demonstrated by the reported studies using various techniques such as IHC, IF, and WB, in multiple brain regions such as the perilesional and ipsilateral cortex, the ipsilateral hippocampus, and the contralateral cortex.

p62 is a protein that targets ubiquitinated proteins and allows their phagocytosis during the macroautophagy (Forde and Dale, 2007; Lamark et al., 2009) that gave contrasting result in literature. In particular, using WB and IF in the brain of TBI mice models, Sarkar et al. (2014) found an increase of the protein between 1 h and 7 days after trauma in the ipsilateral cortex and hippocampus without significant alteration of mRNA in real time-PCR. Besides, Sebastiani et al. (2017) found a decrease of the protein between 1 and 5 days after the trauma using a WB.

Additionally, a recent study from Lang et al. (2024) using brain biopsies from patients with recent TBI, revealed an increase in neurons positive for LC3B staining via immunofluorescence staining compared to controls, as well as an increase in LC3B, Beclin 1, p62, and Atg5 detected by IHC and WB in traumatic brain tissue. Furthermore, LC3B and p62 exhibited a time-dependent increase after injury.

Finally, LAMP2A is an integral lysosome membrane protein involved in CMA process since they bind misfolded protein that are bound to heat shock protein 79 (HSC79) and allow their phagocytosis (Rusten and Stenmark, 2010). Park et al. (2015) describe an elevation LAMP2A both in neurons and glial cells of CA3 and Cx hippocampus areas of the brain bilaterally between 1 and 15 days in a TBI in mice models studied with electrophoresis and IHC. However, Saykally et al. (2018) found a decrease of LAMP2A in the brains of TBI mice models studied with radioimmunoprecipitation 60 days after the trauma.

Since the autophagy process is proven to be active in the TBI process (Clark et al., 2008; Wu and Lipinski, 2019; Zeng et al., 2020; Lang et al., 2024), the present study investigates the use of LC3B, Beclin 1, p62, and LAMP2A as a forensic diagnostic marker for brain trauma.

Interestingly, our data evidence that for LC3B staining the comparison of the three groups results in a statistically significant increase in autophagy-positive neurons in subjects who died between 1 h and 32 days after a TBI (Group B) compared to controls (Group C). These findings align with previous reports of autophagy activation in neurons post-TBI in murine models (Erlich et al., 2006; Lai et al., 2008; Liu et al., 2008; Zhang et al., 2008; Sadasivan et al., 2008; Sun et al., 2014; Sebastiani et al., 2017; Zhang et al., 2017) suggesting that LC3B holds promise as a potential diagnostic marker for head trauma in forensic investigations. Furthermore, a statistical significative difference was detected between group B and group A while no statistical difference was observed between Groups C and A. These results support the consistency between our experimental data and the existing literature, which shows that LC3B activation in murine models is detectable after 1 h (Lai et al., 2008; Sebastiani et al., 2017). This finding may be attributed to the insufficient time to activate autophagy pathways in neurons in cases where death occurred shortly after the trauma, contrarily to cases where death occurred hours or days later.

Regarding p62, two distinct staining patterns were observed: a vesicular pattern localized to a single pole of the cytoplasm, potentially indicative of autophagy activation, and a homogeneous non-autophagic pattern. However, statistical analysis between groups revealed no significant differences in the number of neurons exhibiting a p62 autophagic pattern. This is consistent with the conflicting literature on p62 expression levels following TBI in animal models (Sebastiani et al., 2017; Sarkar et al., 2014).

In contrast, Beclin 1 and LAMP2A staining were not evaluated qualitatively, as no morphological differences were detected between groups during preliminary analysis. Specifically, Beclin 1 showed no detectable variations, while LAMP2A staining was observed exclusively in glial cells, with no neuronal staining.

The differences between our conclusions and those obtained in clinical studies (Lang et al., 2024) can be explained based on the different sample used. Since death is defined as the cessation of cerebral function, autophagy activation in neurons during the agonal phase is expected due to the external insult that causes cerebral damage. Our results suggest that, as in clinical samples, LC3B is a useful marker in forensic samples. Contrarily, peri-mortem modifications appear to influence the applicability of Beclin-1 and p62 in autopsy-derived samples.

Given these promising findings about LC3B, further studies are warranted to validate these data. The differential timing of the four proteins expression and the comparison between the staining of glial cells and neurons should also evaluate. For LC3B, for example, this would help to determine whether they correlate with findings in murine models, which indicate early activation of the protein in neurons, followed by activation in glial cells (Erlich et al., 2006; Lai et al., 2008; Liu et al., 2008; Zhang et al., 2008; Sadasivan et al., 2008; Sun et al., 2014; Sebastiani et al., 2017; Zhang et al., 2017). This could enhance not only the diagnosis of TBI but also, potentially, provide insight into how long before death the trauma occurred, aiding in cases of uncertain circumstances. Therefore, combining the time dependent-expression profiles of these four markers could facilitate the development of a panel of autophagy markers that are differentially expressed and specific to various TBI conditions. In particular, difference in the timing of their expression after death, as well as different pattern in neurons and glia should be examined.

Although other methods, such as western blotting, would be helpful to test protein expression levels, and provide quantitative information to implement the prognostic significance, we think that the individuation of an IHC marker is of fundamental importance. This because in the forensic practice, the paraffin embedded specimen is easily accessible and does not require dedicated processing.

Finally, we are aware that the small number of cases analyzed could be a limitation for the study. The limited number of cases is due to the selection criteria that excluded cases with known relevant pathologies, substance abuse, or poor corpse preservation, represents a major limitation of this study. This limitation precluded the analysis of potential influences such as sex, perimortem and postmortem factors, or pre-existing pathologies, although data from literature report a sex-dependent differences in autophagy signaling pathway and on the differential expression of related proteins (Oliván et al., 2014; Shang et al., 2021) as well as other studies emphasize the activation of autophagy in pathological processes, such as neurological disorders (Kobayashi, 2015; Yamamoto et al., 2023; New and Thomas, 2019). Further research is therefore needed to investigate the possible influence on sex-dependent LC3B expression, impact of diseases of the patients and biases due to factors occurring during the agony of the subjects as well as to the preservation of the corpse. Nonetheless, given the strong statistical significance observed, the results of this pilot study should be considered reliable, and suggest LC3B as a solid marker for TBI diagnosis in forensic samples.

## 5 Conclusion

The results of this study revealed a significant difference in the immunohistochemical staining patterns of brain samples using the anti-LC3B antibody between subjects who died between 1 h and 32 days after a TBI and those who died suddenly without major trauma or shortly after the trauma, providing to LC3B a potential in the investigation of TBI. No staining difference of statistical relevance between the groups was found for Beclin 1, p62 and LAMP2A instead. However, we think that further research is needed to confirm these findings, and to evaluate other qualitative and quantitative aspects. This could eventually lay the background to the integration of these methods into forensic pathology for diagnostic purposes and into clinical practice for prognostic evaluation.

## Data Availability

The raw data supporting the conclusions of this article will be made available by the authors, without undue reservation.

## Footnotes

^1^imagej.nih.gov/ij/

^2^ https://imagej.nih.gov/ij/docs/guide/user-guide.pdf

## Supplementary material

Supplementary Figure S1 Technical negative control obtained using the probe without primary antibody with neurons without staining (yellow arrows).

Supplementary Figure S2 Three brain sections stained with anti-Beclin 1 antibody. **(A)** Section from a case of Group A. **(B)** Section from a case of Group B. **(C)** Section from a control sample (group C).

Supplementary Figure S3 Three brain sections stained with anti-LAMP2A antibody. **(A)** Section from a case of Group A. **(B)** Section from a case of Group B. **(C)** Section from a control sample (group C). The staining selectively highlights glial cells (green arrows), while neurons (black arrows) appear unstained.
