# Supplementary figures and images for: Traumatic brain injury and autophagy: a pilot study about the immunohistochemical expression of LC3B, Beclin 1, p62, and LAMP2A in human autoptic samples
Source: Front Mol Neurosci. 2025 Apr 28;18:1562954. doi: 10.3389/fnmol.2025.1562954 (PMC12066584; doi:10.3389/fnmol.2025.1562954)

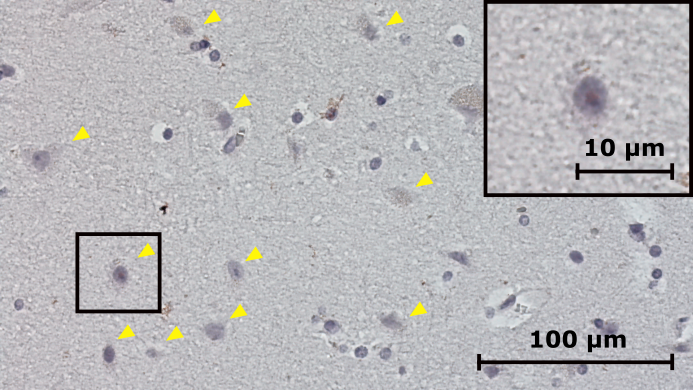

Supplement: Supplementary Figure S1 — Technical negative control obtained using the probe without primary antibody with neurons without staining (yellow arrows). [file Image_1.tiff]

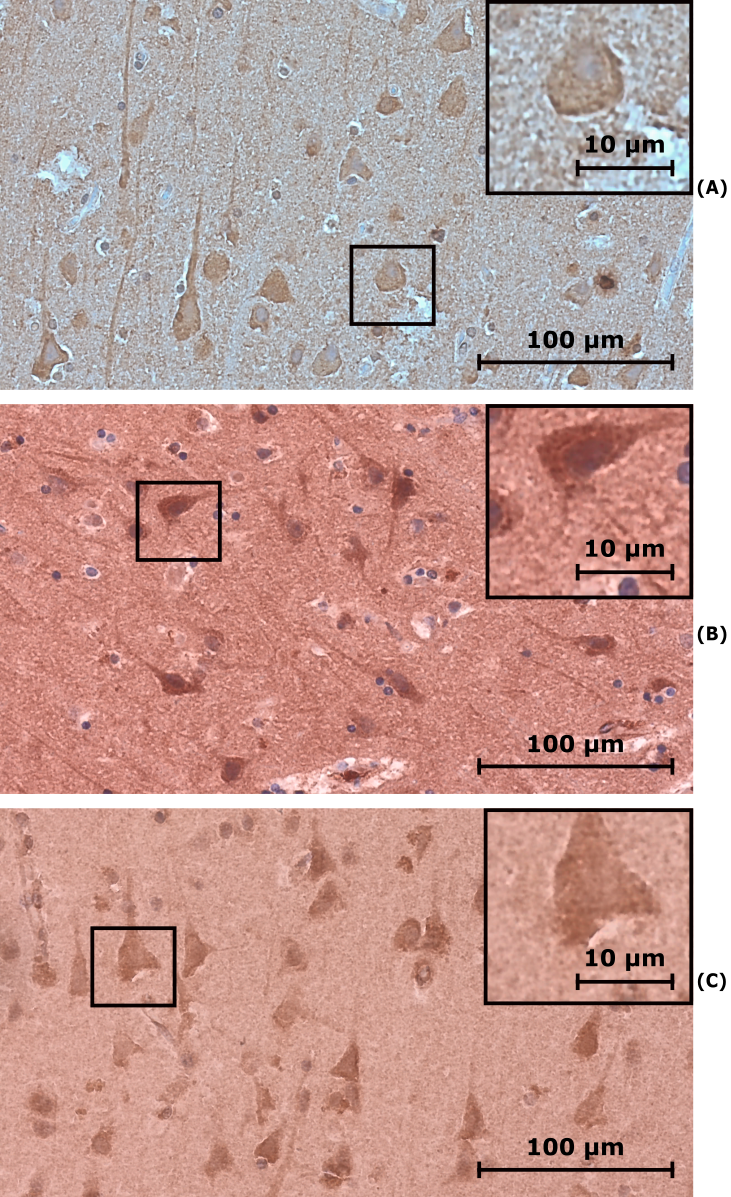

Supplement: Supplementary Figure S2 — Three brain sections stained with anti-Beclin 1 antibody. (A) Section from a case of Group A. (B) Section from a case of Group B. (C) Section from a control sample (group C). [file Image_2.tiff]

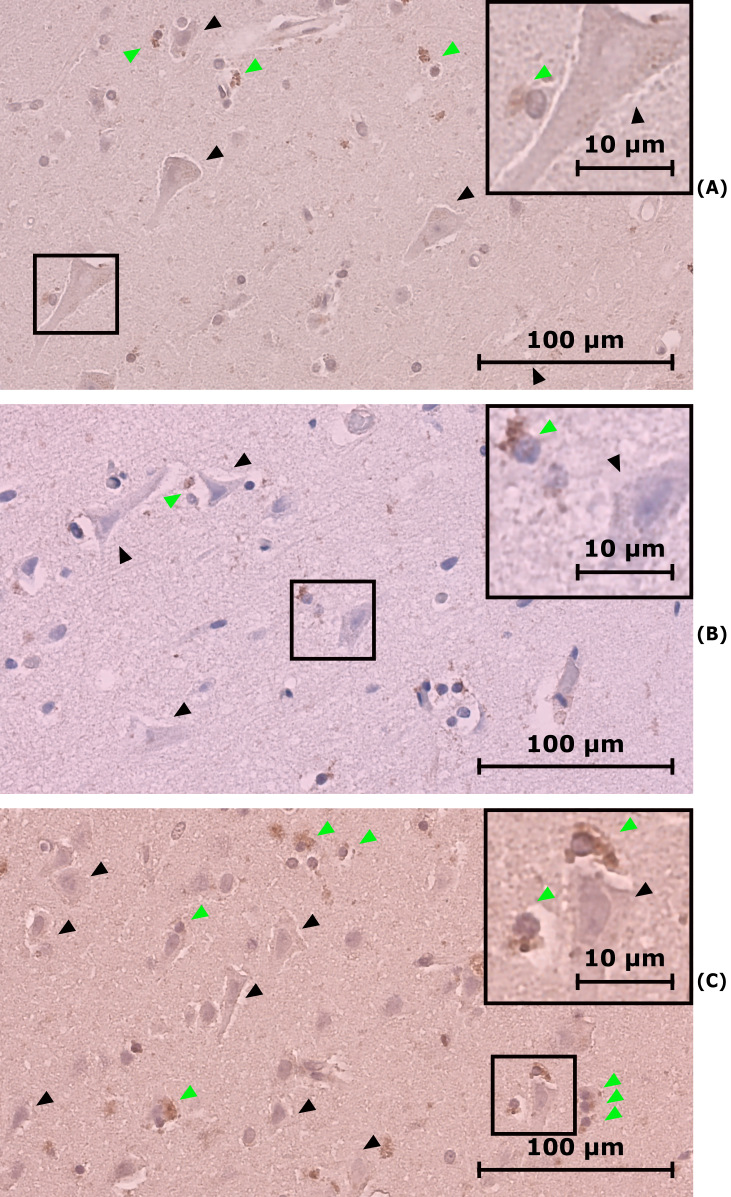

Supplement: Supplementary Figure S3 — Three brain sections stained with anti-LAMP2A antibody. (A) Section from a case of Group A. (B) Section from a case of Group B. (C) Section from a control sample (group C). The staining selectively highlights glial cells (green arrows), while neurons (black arrows) appear unstained. [file Image_3.tiff]
